# Integrating Serological and Genomic Data to Elucidate Lumpy Skin Disease Virus Diversity in Cattle from Bangladesh

**DOI:** 10.3390/v17081126

**Published:** 2025-08-15

**Authors:** Nasrin Sultana Tonu, Sajedul Hayat, Shukes Chandra Badhy, Salima Ferdows, Md. Golam Azam Chowdhury, Babu Kanti Nath, Md Safiul Alam Bhuiyan, Muhammad Jasim Uddin, Suman Das Gupta, Subir Sarker

**Affiliations:** 1 Department of Livestock Services, Ministry of Fisheries and Livestock, Dhaka 1215, Bangladesh; sultanatonu30@gmail.com; 2 Department of Livestock Services, Krishi Khamar Sarak, Dhaka 1215, Bangladesh; sajed.vet@gmail.com; 3 Central Disease Investigation Laboratory, Department of Livestock Services, Krishi Khamar Sarak, Dhaka 1215, Bangladesh; badhy78@gmail.com (S.C.B.); salimaferdows89@gmail.com (S.F.); ga.tulu@yahoo.com (M.G.A.C.); 4 Biosecurity, Gulbali Institute, Charles Sturt University, Wagga Wagga, NSW 2678, Australia; bnath@csu.edu.au (B.K.N.); sgupta@csu.edu.au (S.D.G.); 5 Faculty of Sustainable Agriculture, Livestock Production, University Malaysia Sabah, Locked Bag No. 3, Sandakan 90509, Sabah, Malaysia; md.safiul@ums.edu.my; 6 School of Veterinary Medicine, Murdoch University, Perth, WA 6150, Australia; jasim.uddin@murdoch.edu.au; 7 Centre for Biosecurity and One Health, Harry Butler Institute, Murdoch University, Perth, WA 6150, Australia; 8 School of Agricultural, Environmental and Veterinary Sciences, Faculty of Science and Health, Charles Sturt University, Wagga Wagga, NSW 2678, Australia; 9 Biomedical Sciences & Molecular Biology, College of Medicine and Dentistry, James Cook University, Townsville, QLD 4811, Australia

**Keywords:** lumpy skin disease virus, seroprevalence, whole-genome sequencing, phylogenetics, cattle, emerging transboundary disease

## Abstract

Lumpy skin disease virus (LSDV), a transboundary pathogen threatening cattle health in South and Southeast Asia, presents growing challenges for disease control. This study combined serological, molecular, and genomic approaches to investigate LSDV in Barura Upazila, Bangladesh. Serological screening of 424 cattle using a commercial ELISA revealed a high seroprevalence of 55.5% (95% CI: 50.7–60.3), indicating widespread exposure. Although differences were observed by age and breed, no significant associations were found with seropositivity, suggesting broad regional circulation. Real-time PCR confirmed LSDV DNA in all 20 clinically infected animals, with consistent P32 gene amplification. Two samples with low Cq values underwent whole-genome sequencing. The complete genomes of LSDV-L2/2024 and LSDV-L3/2024 showed >99.6% identity with the reference strain LSDV-29, yet carried unique genomic features, including truncated or variant ORFs and immune-related gene differences. Phylogenetic analysis of the DNA polymerase gene revealed distinct clustering: L2/2024 aligned with South Asian isolates, while L3/2024 grouped with strains from Africa, the Middle East, and Europe. These results highlight co-circulation of genetically diverse strains and possible cross-regional introductions. Overall, our findings underscore the evolutionary plasticity of LSDV and the critical need for ongoing genomic surveillance to guide targeted vaccine development and disease control strategies.

## 1. Introduction

Lumpy skin disease (LSD) is a rapidly emerging, economically devastating viral disease that affects cattle, characterised by cutaneous nodules, systemic illness, and a wide range of secondary complications. It is caused by the lumpy skin disease virus (LSDV), a double-stranded DNA virus belonging to the genus *Capripoxvirus* within the family *Poxviridae* [1]. Though LSD does not pose a zoonotic threat, it is classified by the World Organisation for Animal Health (WOAH) as a notifiable disease due to its potential for rapid transboundary spread and substantial economic impact on livestock production systems. Historically endemic to sub-Saharan Africa, LSD has exhibited significant geographic expansion in recent decades, infiltrating the Middle East, southeastern Europe, and Asia through a combination of climatic, ecological, and anthropogenic factors [2,3,4]. The first confirmed case of LSD in Bangladesh occurred in July 2019 in the Chattogram district, verified by molecular diagnostics and officially reported to WOAH [5,6,7]. Since then, the disease has continued to spread across the country, with increasing frequency and severity of outbreaks.

LSDV harbours a large, linear genome of approximately 150 kilobase pairs, encoding more than 150 open reading frames (ORFs) involved in replication, immune modulation, and virulence [8]. The virus shares high genomic synteny and sequence conservation with other capripoxviruses, including sheeppox virus (SPPV) and goatpox virus (GTPV), yet maintains unique genetic elements that contribute to its host range and transmission dynamics [9,10]. Recent applications of next-generation sequencing (NGS) have facilitated whole-genome analyses of LSDV, revealing insights into strain diversity, recombination events, and molecular epidemiology [11].

The transmission of LSDV is primarily mechanical, mediated by hematophagous arthropod vectors such as mosquitoes (*Aedes*, *Culex*), biting flies (*Stomoxys*, *Tabanus*), and various species of ticks (*Rhipicephalus*, *Amblyomma*) [12,13]. Although direct contact between infected and susceptible animals plays a minor role in transmission, indirect spread via contaminated equipment, vehicles, clothing, and animal handlers has also been implicated. There is emerging evidence that LSDV can be detected in semen and milk, suggesting potential vertical and venereal transmission routes under specific conditions. Environmental factors also significantly influence LSD epidemiology. In Bangladesh, climatic conditions such as high humidity, prolonged monsoon seasons, and marshy terrain create optimal environments for vector proliferation, thereby intensifying transmission dynamics [5,14]. Outbreaks often peak during warmer and wetter months, aligning with increased vector activity.

Clinical signs of LSD in cattle include high fever (40–41.5 °C), anorexia, nasal and ocular discharge, salivation, and marked enlargement of superficial lymph nodes. The hallmark lesions are firm, raised nodules that can coalesce and become necrotic, often accompanied by oedema in the brisket and limb regions [15,16]. While mortality rates are typically low (<10%), morbidity can exceed 70%, especially among naïve or young animals [17,18]. Affected cattle may suffer from reduced feed intake, milk yield, growth rates, and reproductive performance. Complications such as mastitis, orchitis, and abortion can also occur, with long-term impacts on animal health and productivity.

The economic repercussions of LSD are multifaceted, encompassing direct losses due to animal morbidity and indirect costs associated with control measures, diagnostics, vaccination, and trade restrictions. In Bangladesh, the total estimated economic loss from LSD in selected districts has exceeded 90 million USD annually [19]. Similarly, in Ethiopia, herd-level outbreak losses have ranged from USD 489 in subsistence systems to over USD 2700 in commercial operations [20]. These figures underscore the disproportionate impact on smallholder farmers, who depend heavily on livestock for food security, income, and agricultural labour. Additionally, effective disease control requires timely diagnosis, vector control, movement restrictions, and strategic vaccination campaigns. Diagnostic confirmation of LSD typically involves real-time PCR, virus isolation, and serological assays targeting capripoxvirus-specific antibodies [21,22]. Despite ongoing research, challenges remain in differentiating field strains from vaccine-derived viruses, complicating surveillance and outbreak tracking efforts [23,24].

In this context, the present study investigates the molecular epidemiology and genomic architecture of LSDV strains collected during recent outbreaks in Bangladesh. Through serological screening, real-time PCR detection, and whole-genome sequencing, we aim to elucidate the seroprevalence of circulating LSDV and its genetic diversity. Comparative genomic and phylogenetic analyses were performed to position the local strains in a broader evolutionary context and identify lineage-specific features with potential implications for virulence and diagnostics.

## 2. Materials and Methods

### 2.1. Sampling for ELISA

Between July and September 2024, a total of 424 blood samples were aseptically collected from cattle in Barura Upazila, Bangladesh, by a licenced veterinarian. As the samples were collected as part of routine surveillance, animal ethics approval was not required. After collection, the blood was processed to separate serum, which was immediately stored in a cool box and transported to the Central Disease Investigation Laboratory (CDIL) in Dhaka. Upon arrival, the serum samples were preserved at −80 °C until serological testing. Serological analysis was performed using the ID Screen^®^ Capripox Double Antigen Multi-species ELISA kit (IDVet, Rue Louis Pasteur, Grabels, France), following the manufacturer’s recommended procedure. In brief, 50 µL of dilution buffer (buffer 19) was added to each well, followed by 50 µL of test sera, positive control, or negative control. The microplates were incubated at 21 °C (±5 °C) for 90 (±5) min. After incubation, the wells were washed five times with the supplied wash buffer to remove unbound material. Subsequently, 100 µL of conjugate was added to each well, and the plates were incubated again at 21 °C (±5 °C) for 30 (±3) min. A second washing step was performed (five times), after which 100 µL of substrate solution was added to each well. The plates were incubated in the dark at 21 °C (±5 °C) for 15 (±2) min. The reaction was stopped by adding 100 µL of stop solution, and optical density (OD) was measured at 450 nm using a microplate reader. The OD values were then used to calculate the Sample-to-Positive ratio (S/P%) using the following formula:

$$S/P\%\;=\;\frac{\mathrm{OD}\;\mathrm{sample}-\mathrm{OD}\;\mathrm{Negative}}{\mathrm{OD}\;\mathrm{positive}-\mathrm{OD}\;\mathrm{negative}}\;\times \;100$$

Result interpretation: Results were interpreted according to the manufacturer’s guidelines. Samples with an S/P% value less than 30% were considered seronegative for LSDV, while those with an S/P% greater than 30% were classified as seropositive for LSDV antibodies.

### 2.2. Data Analyses for Seroprevalence

Laboratory test results were initially entered into Microsoft Excel for Microsoft 365 (Microsoft Corporation, Redmond, WA, USA), where they were coded, cleaned, and checked for consistency. The finalised dataset was then exported to STATA version 14.1 (StataCorp, College Station, TX, USA) for statistical analysis. Animal-level seroprevalence of LSD was calculated with corresponding 95% logit confidence intervals using the -prop- command in STATA, following the method described by Dean and Pagano (2015) [25]. To characterise seroprevalence patterns, results were stratified by key animal-level variables including age group, breed, and sex.

To evaluate potential associations between these animal-level factors and LSD sero-positivity, univariate logistic regression analyses were conducted. Separate models were run for each explanatory variable (age group, breed, and sex) to estimate odds ratios and corresponding 95% confidence intervals. Statistical significance was determined using a *p*-value threshold of less than 0.05.

### 2.3. Sampling and Viral Nucleic Acid Extraction

Of the 424 cattle sampled, 20 animals displaying characteristic clinical signs of LSD (e.g., nodular skin lesions, fever, lymphadenopathy) were selected for molecular testing by PCR. Skin nodule biopsy samples were aseptically collected from 20 cattle and transported to the Central Disease Investigation Laboratory (CDIL) in Dhaka for molecular diagnostics. Since the sampling was part of a routine veterinary disease monitoring programme, separate animal ethics approval was not required. For nucleic acid extraction, standard protocols were followed [26]. Briefly, biopsy tissues were finely minced using a sterile scalpel and further homogenised using a mortar and pestle. Approximately 10 mL of sterile phosphate-buffered saline (PBS) was added to each homogenate in sterile tubes to facilitate suspension. The homogenates were centrifuged at 1000× *g*, and 200 μL of the resulting supernatant was transferred into Eppendorf tubes for DNA extraction. Genomic DNA was extracted using the GeneJET™ Genomic DNA Purification Kit (Thermo Fisher Scientific Baltics UAB, Vilnius, Lithuania) in accordance with the manufacturer’s instructions. The final elution was carried out with 70 μL of elution buffer, and the purified DNA was stored at −80 °C until further analysis.

### 2.4. Molecular Identification of LSDV by PCR

TaqMan probe-based qPCR for the detection of capripoxvirus DNA was performed as previously described; primers CaPV-074F1 5′-AAAACGGTATATGGAATAGAGTTGGAA-3′ and CaPV-074R1 5′-AAATGAAACCAATGGATGGGATA-3′ were used in conjunction with the black hole Quencher 1 (BHQ1) TaqMan probe CaPV-074P1 5′-6FAM-TGGCTCATAGATTTCCT-BHQ1-3′ [27]. Briefly, a 25 μL volume of the PCR reaction mixture was set up, containing 12.5 μL of the GoTaq^®^ qPCR Master Mix (Promega, Madison, WI, USA), 400 nM of forward and reverse primers, 250 nM of the fluorogenic probe, and 5 μL of template. The reaction was performed using the CFX Opus 96 real-time PCR detection system (Bio-Rad, Hercules, CA, USA) with a thermal profile consisting of an initial denaturation step at 95 °C for 10 min, followed by 45 cycles at 95 °C for 15 s and 60 °C for 60 s, with the fluorescence recording at the end of the combined annealing elongation step.

PCR was also carried out to detect the P32 gene of capripoxviruses using primer sets from a previous study (forward primer, 5′-CGATTTCCATAAACTAAAG-3′ and reverse primer, 5′-CTAAAATTAGAGAGCTATACTTCTT-3′), which amplify the 390 bp fragment within the gene [28]. PCR was performed using the GoTaq Green Master Mix kit (Promega, Madison, WI, USA) in a reaction volume of 25 μL, containing 12.5 μL 2× master mix, 400 nM of each primer, and 2 μL of template DNA. PCR was performed in a C1000 PCR thermal cycler (Bio-Rad, Hercules, CA, USA), and amplification was conducted with the following programme: initial denaturation at 94 °C for 5 min, 35 cycles of denaturation at 94 °C for 30 s, annealing at 50 °C for 30 s, and extension at 72 °C for 30 s, and a final extension phase at 72 °C for 5 min. The PCR products were visualised with the GelDoc Go Imaging System (Bio-Rad, Hercules, CA, USA) after gel electrophoresis for 45 min on 1.5% agarose, stained with ethidium bromide.

### 2.5. Library Preparation and Sequencing

Genomic DNA extracted from two PCR-positive samples was first evaluated for quality and concentration. High-quality DNA was randomly fragmented using a Covaris ultrasonic disruptor to produce fragments suitable for library preparation. The resulting DNA fragments underwent a standard library construction protocol, including end-repair, 3′ adenylation (A-tailing), adapter ligation, and PCR amplification, followed by size selection and purification to generate the final sequencing libraries. Library quality control was conducted by Novogene (Beijing, China) using two methods: fragment analysis with the Advanced Analytical Technologies, Inc. (AATI, Heidelberg, Germany) system to assess DNA integrity and insert size distribution, and quantitative PCR (qPCR) to determine the effective concentration of each library. Libraries that passed quality checks were pooled based on their quantified concentrations and target sequencing output and subjected to paired-end sequencing (2 × 150 bp) on the Illumina NovaSeq 6000 platform using the PE150 strategy by Novogene (Beijing, China).

### 2.6. Bioinformatic Analyses for LSDV Genome Assembly

Sequencing data were analysed using an established workflow [29,30,31,32,33] implemented in Geneious Prime (version 2023.1.1, Biomatters, New Zealand) and processed on the high-performance computing (HPC) system at James Cook University. The analysis began with an initial quality assessment of raw sequencing reads, followed by preprocessing steps to remove ambiguous base calls, low-quality reads, and Illumina adapter sequences. Trimmed reads were then aligned to the cattle genome (*Bos taurus*, accession no. GCA_002263795.3) to eliminate potential host DNA contamination. To further refine the dataset, reads were subsequently mapped to the *Escherichia coli* genome (GenBank accession no. U00096) to exclude bacterial sequences. The remaining unmapped reads were subjected to de novo assembly using the SPAdes assembler (version 3.10.1) [34], with the ‘careful’ setting to improve assembly accuracy. The resulting contigs were screened against GenBank’s non-redundant nucleotide (BLASTN) and protein (BLASTX) databases [35], applying an E-value threshold of 1 × 10^−5^ to reduce false-positive identifications. Contigs that showed significant similarity to bacterial, eukaryotic, or fungal sequences were discarded, ensuring that only viral sequences were retained for further analysis.

### 2.7. Genome Annotations

The genomes of LSDV-L2 and LSDV-L3 were annotated using Geneious (version 2023.1.1), referencing the LSDV isolate LSD-29 genome from Bangladesh (GenBank accession no. PP746705) [36,37]. Open reading frames (ORFs) exceeding 50 amino acids, possessing an ATG start codon, and exhibiting minimal overlap (≤50%) with adjacent genes were identified and annotated [29]. Additionally, previously annotated ORFs shorter than 50 amino acids from other LSDV genomes were included. These ORFs were extracted into a FASTA file for similarity searches using BLAST (version 2.16.0). ORFs displaying significant sequence similarity to known viral or cellular genes (BLAST E-value ≤ 1 × 10^−5^) were classified as potential genes [35]. Unless specified otherwise, default software parameters were applied.

### 2.8. Comparative Genomics and Phylogenetic Analyses

Comparative genomic analysis of the newly sequenced LSDV genomes was conducted using Geneious Prime (version 2023.1.1). Sequence similarity between the selected LSDV sequences and reference genomes was evaluated through multiple sequence alignment using the MAFFT L-INS-I algorithm, implemented within Geneious Prime (Biomatters, Ltd., Auckland, New Zealand).

Phylogenetic analysis was performed using representative LSDV genomes or conserved gene sequences retrieved from GenBank. Amino acid sequences of selected protein-coding genes were aligned using the MAFFT L-INS-I algorithm within Geneious Prime (version 7.388) [38]. A phylogenetic tree was constructed in Geneious Prime (version 2023.1.1) using RAxML with the Gamma Blosum62 protein model and 1000 bootstrap replicates to ensure robust statistical support. The resulting tree was visualised with FigTree v1.4.4 for interpretation and presentation.

## 3. Results

### 3.1. Seroprevalence of LSDV

**Figure 1:**
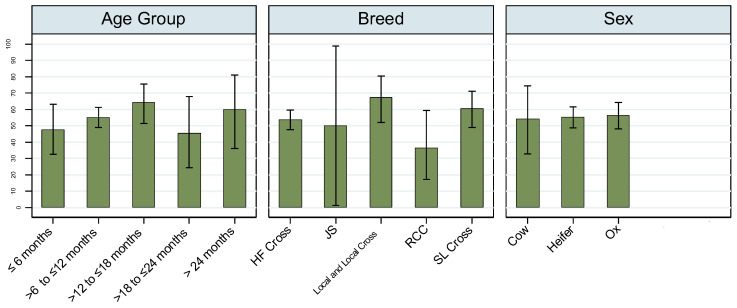
Animal-level lumpy skin disease (LSD) seroprevalence by age group, breed, and sex of cattle in the study population. Bars represent the proportion of seropositive animals within each category, and vertical lines indicate 95% confidence intervals.

The overall animal-level seroprevalence of LSD was 55.5% (95% CI: 50.7–60.3). Among age groups, the highest seroprevalence was observed in cattle aged >12 to ≤18 months (64.2%, 95% CI: 51.5–75.5), while lower prevalence was noted in the ≤6 months (47.7%, 95% CI: 32.5–63.3) and >18 to ≤24 months (45.5%, 95% CI: 24.4–67.8) groups. Regarding breed, Local and Local Cross cattle showed the highest seropositivity (67.4%, 95% CI: 52.0–80.5), whereas Red Chittagong Cattle (RCC) had the lowest (36.4%, 95% CI: 17.2–59.3). Seroprevalence by sex was relatively consistent across cows (54.2%, 95% CI: 32.8–74.4), heifers (55.2%, 95% CI: 48.8–61.5), and oxen (56.3%, 95% CI: 48.0–64.3) (Figure 1).

Although descriptive trends suggested higher seroprevalence in specific age and breed groups, univariate logistic regression analyses did not identify any statistically significant associations. Specifically, no significant relationship was found between LSD seropositivity and age group, breed, or sex (Supplementary Table S1). These findings suggest that, within this study population, animal-level factors such as age, breed, and sex were not significant predictors of LSD exposure based on serological evidence.

### 3.2. Molecular Detection of LSDV

Real-time PCR testing identified the presence of LSDV DNA in all 20 cattle samples analysed, confirming active viral infection. Each sample, collected from the Barura Upazila region, produced a 390 base pair amplicon targeting the P32 gene, as visualised through gel electrophoresis. The consistent detection of this fragment across all samples indicates a strong likelihood of LSDV being the causative agent, as the P32 gene is a conserved marker within the *Capripoxvirus* genus.

To enable more detailed genetic analysis, two samples with the lowest Cq values—15.27 and 17.63—were selected for whole-genome sequencing. These low Cq values are indicative of high viral DNA concentration, which improves the chances of obtaining complete and high-quality genomic sequences.

### 3.3. Genome Sequences of LSDV Strains L2 and L3

The fully assembled genomes of LSDV strain L2/2024 (LSDV-L2) and L3/2024 (LSDV-L3) were determined to be linear double-stranded DNA molecules, consisting of 150,643 and 151,130 nucleotides, respectively. These sequences have been deposited in GenBank under accession numbers PV066181–PV066182. Similarly to other poxviruses [29,36,39,40,41,42], the LSDV-L2 and LSDV-L3 genomes contain a large central coding region flanked by two identical inverted terminal repeats (ITRs), measuring 2354 bp (coordinates 1–2354 in the sense strand and 148,290–150,643 in the antisense orientation) for LSDV-L2, and 2609 bp (coordinates 1–2609 in the sense strand and 148,522–151,130 in the antisense orientation) for LSDV-L3. Comparative genomic analysis revealed that LSDV-L2 and LSDV-L3 share the highest nucleotide identity (99.73% and 99.61%, respectively) with the LSDV isolate LSD-29 from cattle in Bangladesh (GenBank accession no. PP746705) [36].

### 3.4. Genome Annotation and Comparative Analyses

The genomes of LSDV-L2 and LSDV-L3 contained 162 and 163 predicted open reading frames (ORFs) with methionine start codons, respectively, which were annotated as putative genes and numbered sequentially from left to right (Supplementary Tables S2 and S3). Of these, four ORFs in each genome were located within the inverted terminal repeats (ITRs) and were thus present as diploid copies. Comparative analysis of the predicted ORFs revealed that, aside from two ORFs within the ITRs of both genomes, all other ORFs exhibited homology with previously identified LSDV gene products (Supplementary Tables S2 and S3).

The conserved genes of LSDV-L2 and LSDV-L3 displayed the highest sequence similarity to homologs of the LSDV-LSD-29 isolate from Bangladesh, suggesting a shared evolutionary lineage. Compared to LSDV isolate LSD-29, LSDV-L2, and LSDV-L3 harboured two additional predicted protein-coding genes (ORF001 and ORF162 in LSDV-L2; ORF001 and ORF163 in LSDV-L3) that were absent in other poxviruses and showed no matches in the NR protein database when analysed using BLASTX and BLASTP (Supplementary Tables S2 and S3).

To characterise the genetic variability among the newly identified LSDV strains, we conducted whole-genome comparisons against the reference strain LSDV-29 and the vaccine-associated strain LSDV-29A. Although the overall genome architecture and nucleotide identity remained largely conserved, several notable differences were observed across the genomes of strains L2/2024 and L3/2024. In strain L2/2024, a single ORF was found to be absent when compared to the LSDV-29 genome (Table 1). In addition, relative to LSDV-29A, strains L2/2024 and L3/2024 were predicted to possess two and three additional ORFs, respectively. Both strains also exhibited a number of ORFs that were either truncated or extended: nine in L2/2024 and ten in L3/2024. Among these, a gene encoding the conserved mRNA decapping enzyme appeared to be fragmented in L3/2024, potentially affecting post-transcriptional regulation during infection. Furthermore, a second conserved gene, annotated as a putative E3 ubiquitin ligase, was found to be truncated in both L2/2024 and L3/2024 compared to LSDV-29.

### 3.5. Evolutionary Relationships of LSDV Sequenced in This Study

**Figure 2:**
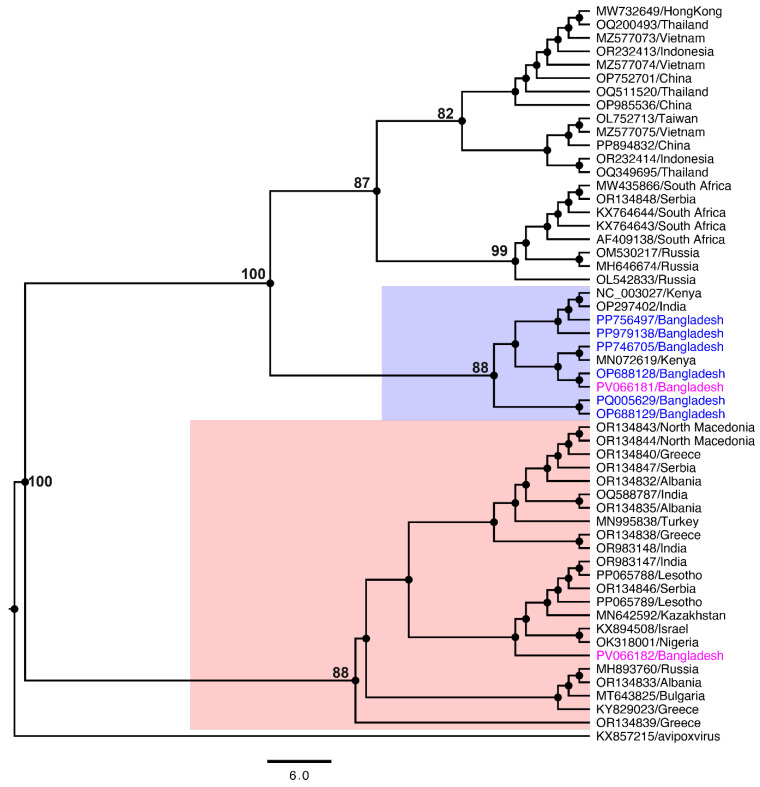
A phylogenetic tree depicting the evolutionary relationships among selected LSDV isolates. Complete DNA polymerase gene sequences were extracted from representative LSDV strains across various countries and aligned using MAFFT (v7.450) with the G–INS–I algorithm (BLOSUM62 scoring matrix; gap open penalty: 1.53; offset value: 0.123) in Geneious Prime (v23.1.1, Biomatters, Ltd., Auckland, New Zealand). A maximum likelihood (ML) tree was constructed in Geneious Prime with 1000 bootstrap replicates, using an avipoxvirus sequence (GenBank accession no. KX857215) as the outgroup. The branch tip labels indicate GenBank accession numbers followed by the country of origin. LSDVs sequenced in this study and previously reported Bangladeshi isolates are highlighted in pink and blue, respectively. The tree was visualised using FigTree v1.4.4, with an automatic scale bar added. Bootstrap values are displayed as percentages, with values below 80% omitted.

Phylogenetic analysis based on the nucleotide sequences of the entire DNA polymerase gene of LSDV revealed that the strains sequenced in this study form two distinct subclades within a larger lineage, primarily comprising isolates from Bangladesh and neighbouring regions. Notably, the LSDV strain L2/2024 clustered predominantly with Bangladeshi isolates, alongside strains sequenced from India and Kenya, supported by a strong bootstrap value (>87%) (Figure 2). This close genetic relationship suggests a common evolutionary lineage among these sequences, potentially reflecting regional transmission patterns or shared epidemiological origins.

In contrast, the LSDV strain L3/2024, sequenced in this study, formed a separate clade within a broader subclade encompassing isolates from Lesotho, India, multiple Middle Eastern countries, Africa, and Europe. Despite their close genetic relatedness, as evidenced by their placement within the same overarching clade, the divergence between L2/2024 and L3/2024 suggests that they may have originated from a highly similar ancestral strain but have since undergone regional diversification.

The phylogenetic clustering of L2/2024 with isolates from Bangladesh, India, and Kenya, as well as the distinct grouping of L3/2024 with a more geographically diverse set of isolates, underscores the complex transmission dynamics of LSDV. These findings highlight the potential role of cross-border livestock movement and environmental factors in shaping the virus’s genetic diversity and epidemiological spread in South Asia and beyond.

## 4. Discussion

The present study provides an evaluation of LSDV seroprevalence in a naturally infected cattle population in Barura Upazila, Bangladesh. The integration of serological, molecular, and genomic tools allowed us to characterise both the extent of virus exposure and the diversity of circulating strains, offering a more nuanced understanding of LSDV dynamics in an endemic context.

The observed seroprevalence of 55.5% indicates substantial exposure within the cattle population. While variations were noted across age groups and breeds—particularly higher seropositivity among cattle aged >12 to ≤18 months (64.2%) and local crossbred animals (67.4%)—these associations were not statistically significant. But these trends are worthy of biological consideration. Cattle in the >12 to ≤18-month age category would typically be experiencing a transition from passive maternal immunity and early-stage protection towards more independent grazing behaviour, perhaps increasing their exposure to competent vectors and ticks. The waning of maternal antibodies at this stage might also increase their immunological susceptibility. Moreover, this age category might be faced with some stressors such as weaning or management variation, which would influence immune competence transiently. Similarly, indigenous and crossbred cattle, under more extensive systems with greater outdoor exposure and limited vector control measures, could be at greater risk of infection. This lack of differentiation suggests a widespread risk of exposure across the population, potentially driven by ecological and vector-borne transmission pressures rather than host-level susceptibility. Similar high levels of seroprevalence have been reported in other endemic countries, including Ethiopia and Jordan, where vector ecology and regional livestock movements significantly influence disease spread [43,44,45]. While serology testing provided valuable measures of widespread exposure, it is incapable of differentiating between recent and previous infection or vaccine-induced and natural infection of LSDV. To address this limitation, molecular and genomic methods were applied to define circulating strains, enabling more precise information on virus evolution and diversity within the region.

Whole-genome sequencing of two PCR positive samples revealed extensive similarity with previously characterised strains, notably the Bangladeshi isolate LSDV-29. However, both LSDV-L2/2024 and LSDV-L3/2024 exhibited subtle yet important genomic differences. These included the presence of unique and truncated ORFs, particularly in regions known to influence host–pathogen interactions. For instance, alterations in genes predicted to encode mRNA decapping enzymes or E3 ubiquitin ligases may have consequences for viral replication efficiency or immune modulation, as such functions are critical in poxvirus biology [46,47,48,49].

Phylogenetic analysis showed that LSDV-L2/2024 was more closely related to strains circulating in South Asia and East Africa, forming a cluster with isolates from Bangladesh, India, and Kenya. In contrast, LSDV-L3/2024 grouped with a broader clade containing strains from Europe, the Middle East, and southern Africa. The phylogenetic separation between these two strains, despite their co-occurrence in a confined region, suggests multiple sources of introduction or the ongoing parallel evolution of diverse viral lineages. These patterns may reflect the influence of animal trade routes, vaccine usage, or undocumented outbreaks—mechanisms previously implicated in the global spread of LSDV [50,51,52,53]. Moreover, the coexistence of divergent strains in a single geographic setting raises important implications for disease management. Vaccines currently in use, typically based on live attenuated strains, may not provide uniform protection across genetically diverse LSDV variants. Emerging studies have raised concerns about vaccine escape, recombination between field and vaccine strains, and incomplete cross-protection, particularly where multiple lineages co-circulate [52,54,55,56]. While our data do not suggest recombination in the sequenced genomes, the presence of ORFs with no known homology and truncations in genes related to host immune evasion merit further functional investigation. Additionally, considering the regional livestock movement, ecological overlap of vector species, and growing risk of re-introduction or novel strain emergence, genomics-based monitoring should be integrated into routine surveillance systems.

The first limitation of this study is that the ELISA used in this study cannot distinguish between antibodies generated through natural infection and those induced by vaccination. As vaccination history was not recorded for all sampled animals, we could not stratify seropositivity by vaccination status. Future investigations should incorporate detailed vaccination records to enable more accurate interpretation of serological data and differentiation between infection- and vaccine-induced immunity. Another limitation of this study is the modest number of whole genomes sequenced, which may not fully capture the range of viral diversity present across broader regions or among asymptomatic animals. Only 2 of the 20 PCR-positive samples were subjected to WGS, as these had the lowest Cq values, ensuring sufficient nucleic acid for near-complete genome recovery with our protocol. This selection maximised sequencing success but may bias results toward high-viral-load cases, underrepresenting viral diversity in lower-load infections. Future studies should apply targeted enrichment, increase input volumes, or perform deeper sequencing to recover genomes from higher-Cq samples and expand spatio-temporal sampling and functional studies to determine how identified genomic differences influence virulence, transmission, or vaccine responsiveness.

## 5. Conclusions

This study provides critical insight into the seroprevalence and genomic landscape of LSDV in a specific region of Bangladesh. The high seroprevalence observed among cattle indicates widespread prior exposure, underscoring the endemic nature of LSD in the region. Molecular confirmation of active infection and whole-genome sequencing of field isolates revealed the co-circulation of genetically distinct LSDV strains, reflecting complex transmission dynamics likely influenced by regional animal movements and vector ecology. Genomic comparisons highlighted both conserved and unique features in the newly sequenced strains, including the presence of additional ORFs, gene truncations, and polymorphisms potentially associated with viral replication and immune evasion. Phylogenetic analysis revealed close evolutionary relationships with strains from South Asia and Africa, as well as clustering patterns suggestive of multiple introduction events or parallel viral evolution. These findings collectively highlight the urgent need for enhanced genomic surveillance, updated diagnostic tools capable of differentiating field and vaccine strains, and adaptive control strategies tailored to local epidemiological realities. In regions where LSDV is endemic or emerging, such integrated molecular approaches will be essential for guiding vaccination policies, monitoring viral evolution, and preventing transboundary spread

## Figures and Tables

**Table 1:** ORFs of interest in the LSDV genome identified in this study compared to a previously identified LSDV genome from Bangladesh.

ORFs	Genome Coordinate (Nucleotide Length); Isolate LSD-29; GenBank Accession No. PP746705	Genome Coordinate (Nucleotide Length); Strain L2/2024; GenBank Accession No. PV066181	Genome Coordinate (Nucleotide Length); Strain L3/2024; GenBank Accession No. PV066182	Notes
Hypothetical protein	-	351–76 (276)	326–60 (267)	No significant identity to known ORFs in GenBank: unique to L2 and L3
Hypothetical protein	2028–1306 (723)	2330–1518 (813)	2215–1493 (723)	N-terminus extended due to deletion/insertion
Putative E3 ubiquitin ligase	6806–6312 (495)	6865–6377 (489)	7122–6634 (489)	L2 and L3: C-terminus shortened due to deletion/insertion
Kelch-like protein	13,665–12,856 (810)	13,724–12,915 (810)	13,971–13,276 (696)	L3: C-terminus shortened due to deletion/insertion
Pox F11 superfamily protein	18,369–17,908 (462)	18,428–17,967 (462)	18,666–18,328 (339)	L3: C-terminus shortened due to deletion
Pox F11 superfamily protein	18,815–18,492 (324)	18,875–17,967 (909)	19,113–18,790 (324)	L2: N-terminus extended due to deletion/insertion
mRNA decapping enzyme	80,411–81,172 (762)	80,471–81,232) (762)	80,691–81,023 (333) 81,187–81,453 (267)	Fragmented in L3 due to deletion/insertion
Kelch-like protein	135,407–136,219 (813)	135,466–136,278 (813)	135,694–137,346 (1653)	L3: C-terminus extended due to deletion/insertion
Kelch-like protein	136,210–137,052 (843)	136,269–137,111 (843)	136,495–137,346 (852)	L3: C-terminus extended due to deletion/insertion
Hypothetical protein	148,154–148,429 (276)	148,212–149,126 (915)	148,444–148,698 (255)	L2: C-terminus extended due to deletion/insertion
Hypothetical protein	148,499–149,221 (723)	-	148,916–149,638 (723)	L2: missing
Hypothetical protein	-	150,293–150,568 (276)	150,805–151,071 (267)	No significant identity to known ORFs in GenBank: unique to L2 and L3

## Data Availability

Nucleotide sequences and associated data from this study are available in the DDBJ/EMBL/GenBank databases under accession numbers PV066181-PV066182.

## Supplementary Materials

The following supporting information can be downloaded at: https://www.mdpi.com/article/10.3390/v17081126/s1, Table S1: Univariate logistic regression results assessing associations between animal-level factors and LSD seroprevalence; Table S2: Open reading frames of LSDV (L2/2024); Table S3: Open reading frames of LSDV (L3/2024).
