# Supplementary material for: Integrating Serological and Genomic Data to Elucidate Lumpy Skin Disease Virus Diversity in Cattle from Bangladesh
Source: Viruses. 2025 Aug 15;17(8):1126. doi: 10.3390/v17081126 (PMC12390663; doi:10.3390/v17081126)
Supplement: Supplementary file 1 [file viruses-17-01126-s001.zip › viruses-3799869-supplementary.pdf]

Supplementary file

Integrating Serological and Genomic Data to Elucidate Lumpy Skin Disease Virus Diversity in Cattle from Bangladesh

Nasrin Sultana Tonu[1], Sajedul Hayat[2], Shukes Chandra Badhy[3], Salima Ferdows[3], Md. Golam Azam Chowdhury[3], Babu Kanti Nath[4], Md Safiul Alam Bhuiyan[5], Md. Jasim Uddin[6,7], Suman Das Gupta[4,8], Subir Sarker[9,*]

[1] Department of Livestock Services, Ministry of Fisheries and Livestock, Bangladesh. Email: sultanatonu30@gmail.com
[2]Department of Livestock Services, Bangladesh. Email: sajed.vet@gmail.com
[3]Central Disease Investigation Laboratory, Department of Livestock Services, Bangladesh. Email: S.C.B., badhy78@gmail.com; S.F., salimaferdows89@gmail.com; G.A.C., ga.tulu@yahoo.com
[4]Biosecurity, Gulbali Institute, Charles Sturt University, Wagga Wagga, NSW 2678, Australia. Email: B.K.N., bnath@csu.edu.au.
[5]Faculty of Sustainable Agriculture, Livestock Production, University Malaysia Sabah, Locked Bag No. 3, Sandakan 90509, Sabah, Malaysia. Email: md.safiul@ums.edu.my
[6]School of Veterinary Medicine, Murdoch University, Perth, WA 6150, Australia
[7]Centre for Biosecurity and One Health, Harry Butler Institute, Murdoch University, Perth, WA 6150, Australia. Email: M.J.U., jasim.uddin@murdoch.edu.au
[8]School of Agricultural, Environmental and Veterinary Sciences, Faculty of Science and Health, Charles Sturt University, Wagga Wagga, NSW-2678, Australia. Email: S.D.G., sgupta@csu.edu.au
[9]Biomedical Sciences & Molecular Biology, College of Medicine and Dentistry, James Cook University, Townsville, QLD 4811, Australia. Email: subir.sarker@jcu.edu.au

*Correspondence: subir.sarker@jcu.edu.au

Supplementary Table S1: Univariate logistic regression results assessing associations between animal-level factors and LSD seroprevalence.

Predictor	Category	Positive % (n)	Negative % (n)	OR (95% CI)	P value
Breed	Holstein Friesian Cross	53.6 (147)	46.4 (127)	Reference	0.1413
	Jersey Cross	50.0 (1)	50.0 (1)	0.9 (0.1-14.0)	
	Local Breed and Local Cross	67.4 (31)	32.6 (15)	1.8 (0.9-3.5)	
	Red Chittagong Cattle (RCC??)	36.4 (8)	63.6 (14)	0.5 (0.2-1.2)	
	Shahiwal Cross	60.5 (49)	39.5 (32)	1.3 (0.8-2.2)	
Age group	≤6 months	47.7 (21)	52.3 (23)	Reference	0.3857
	>6 to ≤12 months	55.2 (150)	44.8 (122)	1.3 (0.7-2.5)	
	>12 to ≤18 months	64.2 (43)	35.8 (24)	2.0 (0.9-4.3)	
	>18 to ≤24 months	45.5 (10)	54.5 (12)	0.9 (0.3-2.5)	
	>24 months	60.0 (12)	40.0 (8)	1.6 (0.6-4.8)	
Sex	Cow	54.2 (13)	45.8 (11)	Reference	0.9683
	Heifer	55.2 (138)	44.8 (112)	1.0 (0.4- 2.4)	
	Ox	56.3 (85)	43.7 (66)	1.1 (0.5-2.6)	

Supplementary Table 1: Open reading frames of LSDV (L2/2024)

ORF (LSDV_L2/2024)	ORF (LSDV-isolate LSD-29)	Product	Genome coordinate_LSDV_L2/2024	Nucleotide	AA	% Identity	Note
ORF1		hypothetical protein	351-76	276	91	-	Unique
ORF2	ORF1	hypothetical protein	802-323	480	159	100	
ORF3	ORF2	hypothetical protein	1268-873	396	131	100	
ORF4	ORF3	hypothetical protein	2330-1518	813	270	90.8	
ORF5	ORF4	IL-10-like protein	2382-2894	513	170	99.4	
ORF6	ORF5	putative IL-1-beta-inhibitor	3600-2905	696	231	100	
ORF7	ORF6	putative IL-1 receptor antagonist	4689-3622	1068	355	100	
ORF8	ORF7	soluble interferon-gamma receptor-like protein	5600-4773	828	275	100	
ORF9	ORF8	hypothetical protein	6325-5633	693	230	100	
ORF10	ORF9	putative E3 ubiquitin ligase	6865-6377	489	162	98.8	
ORF11	ORF10	putative G-protein coupled chemokine receptor	8054-6909	1146	381	100	
ORF12	ORF11	ankyrin repeat protein	8795-8160	636	211	100	
ORF13	ORF12	IL-1 receptor-like protein	9859-8834	1026	341	100	
ORF14	ORF13	eIF2alpha-like PKR inhibitor	10188-9919	270	89	100	
ORF15	ORF14	putative IL-18 binding protein	10660-10175	486	161	100	

ORF16	ORF15	EGF-like growth factor	10966-10697	270	89	100	
ORF17	ORF16	antiapoptotic protein	11487-10957	531	176	100	
ORF18	ORF17	dUTPase	11969-11529	441	146	100	
ORF19	ORF18	kelch-like protein	12888-12016	873	290	100	
ORF20	ORF19	kelch-like protein	13724-12915	810	269	100	
ORF21	ORF20	ribonucleotide reductase small subunit	14754-13789	966	321	100	
ORF22	ORF21	hypothetical protein	15055-14795	261	86	100	
ORF23	ORF22	hypothetical protein	15434-15096	339	112	100	
ORF24	ORF23	hypothetical protein	15883-15665	219	72	100	
ORF25	ORF24	S-S bond formation pathway protein	16610-15960	651	216	100	
ORF26	ORF25	putative Ser|Thr protein kinase	17931-16588	1344	447	100	
ORF27	ORF26	Pox F11 superfamily protein	18428-17967	462	153	100	
ORF28	ORF27	Pox F11 superfamily protein	18875-17967	909	302	35.4	
ORF29	ORF28	hypothetical protein	20800-18884	1917	638	100	
ORF30	ORF29	palmitylated EEV membrane protein	21919-20807	1113	370	100	
ORF31	ORF30	hypothetical protein	22092-21946	147	48	100	
ORF32	ORF31	Pox F15 superfamily protein	22558-22121	438	145	100	
ORF33	ORF32	Pox F16 superfamily protein	23294-22635	660	219	100	

ORF34	ORF33	putative DNA-binding phosphoprotein	23369-23683	315	104	100	
ORF35	ORF34	poly-A polymerase catalytic subunit	25111-23687	1425	474	100	
ORF36	ORF35	hypothetical protein	27315-25108	2208	735	100	
ORF37	ORF36	double-stranded RNA binding protein	27860-27327	534	177	100	
ORF38	ORF37	DNA-dependent RNA polymerase subunit	28526-27921	606	201	100	
ORF39	ORF38	abundant component of virosome	28609-29733	1125	374	100	
ORF40	ORF39	hypothetical protein	29742-31442	1701	566	100	
ORF41	ORF40	membrane protein	31449-32249	801	266	100	
ORF42	ORF41	DNA polymerase	35278-32246	3033	1010	100	
ORF43	ORF42	sulfhydryl oxidase	35312-35599	288	95	100	
ORF44	ORF43	virion core protein	35988-35596	393	130	100	
ORF45	ORF44	hypothetical protein	38029-35975	2055	684	100	
ORF46	ORF45	DNA-binding core protein	39079-38135	945	314	100	
ORF47	ORF46	viral morphogenesis protein	39304-39086	219	72	100	
ORF48	ORF47	ssDNA-binding phosphoprotein	40135-39305	831	276	100	
ORF49	ORF48	IMV protein	40417-40181	237	78	100	
ORF50	ORF49	telomere mismatch binding protein	41619-40435	1185	394	100	
ORF51	ORF50	viral core cysteine proteinase	42913-41612	1302	433	100	

ORF52	ORF51	RNA-helicase	42919-44949	2031	676	100	
ORF53	ORF52	putative metalloproteinase	46736-44946	1791	596	100	
ORF54	ORF53	entry-fusion complex component	47065-46733	333	110	100	
ORF55	ORF54	late transcription elongation factor	47059-47727	669	222	100	
ORF56	ORF55	thioredoxin-like protein	48071-47691	381	126	100	
ORF57	ORF56	Fen1-like nuclease	48074-49387	1314	437	100	
ORF58	ORF57	DNA-dependent RNA polymerase subunit	49388-49579	192	63	100	
ORF59	ORF58	hypothetical protein	49579-50103	525	174	100	
ORF60	ORF59	virion structural protein	51238-50117	1122	373	100	
ORF61	ORF60	late transcription factor	51268-52050	783	260	100	
ORF62	ORF61	myristylprotein	52077-53087	1011	336	100	
ORF63	ORF62	IMV membrane protein	53088-53825	738	245	100	
ORF64	ORF63	crescent formation protein	53863-54141	279	92	100	
ORF65	ORF64	early transcription protein	55107-54151	957	318	100	
ORF66	ORF65	core protein	55132-55893	762	253	100	
ORF67	ORF66	putative membrane protein	55909-56304	396	131	100	
ORF68	ORF67	virion protein	56261-56704	444	147	100	
ORF69	ORF68	thymidine kinase	56732-57265	534	177	100	

ORF70	ORF69	host-range protein	57337-57933	597	198	100	
ORF71	ORF70	multifunctional poly-A polymerase regulatory subunit	57991-58992	1002	333	100	
ORF72	ORF71	DNA-dependent RNA polymerase subunit	58907-59464	558	185	100	
ORF73	ORF72	late 16kDa putative membrane protein	59871-59470	402	133	100	
ORF74	ORF73	DNA-dependent RNA polymerase subunit	59957-63814	3858	1285	100	
ORF75	ORF74	Tyr|Ser protein phosphatase	64334-63819	516	171	100	
ORF76	ORF75	entry-fusion complex component	64350-64922	573	190	100	
ORF77	ORF76	IMV heparin binding surface protein	65887-64919	969	322	100	
ORF78	ORF77	hypothetical protein	68313-65917	2397	798	100	
ORF79	ORF78	late transcription factor	68457-69128	672	223	100	
ORF80	ORF79	topoisomerase type IB	69170-70123	954	317	100	
ORF81	ORF80	virion morphogenesis protein	70143-70586	444	147	100	
ORF82	ORF81	large subunit of mRNA capping enzyme	70617-73145	2529	842	100	
ORF83	ORF82	virion core protein	73574-73107	468	155	100	
ORF84	ORF83	virion core protein	73576-74313	738	245	100	

ORF85	ORF84	uracil-DNA glycosylase	74310-74966	657	218	100	
ORF86	ORF85	NTPase	75009-77369	2361	786	100	
ORF87	ORF86	70kDa small subunit of early transcription factor	77366-79273	1908	635	100	
ORF88	ORF87	DNA-dependent RNA polymerase subunit	79298-79789	492	163	100	
ORF89	ORF88	mRNA decapping enzyme	79830-80471	642	213	100	
ORF90	ORF89	mRNA decapping enzyme	80471-81232	762	253	100	
ORF91	ORF90	ATPase nucleoside triphosphate phosphohydrolase-I	83145-81238	1908	635	100	
ORF92	ORF91	small subunit of mRNA capping enzyme	84035-83172	864	287	100	
ORF93	ORF92	rifampicin target	85724-84075	1650	549	100	
ORF94	ORF93	late transcription factor	86203-85751	453	150	100	
ORF95	ORF94	putative late transcription factor	86931-86233	699	232	100	
ORF96	ORF95	S-S bond formation pathway protein	87155-86928	228	75	100	
ORF97	ORF96	hypothetical protein	89149-87164	1986	661	100	
ORF98	ORF97	putative virion core protein	89759-89274	486	161	100	

ORF99	ORF98	DNA-dependent RNA polymerase subunit	89800-90312	513	170	100	
ORF100	ORF99	virion core protein	91436-90309	1128	375	100	
ORF101	ORF100	82kDa large subunit of early transcription factor	93601-91457	2145	714	100	
ORF102	ORF101	32kDa small subunit of transcription factor	93658-94530	873	290	100	
ORF103	ORF102	IMV membrane protein	94790-94554	237	78	100	
ORF104	ORF103	hypothetical protein	97505-94791	2715	904	100	
ORF105	ORF104	viral membrane formation protein	97520-98473	954	317	100	
ORF106	ORF105	core protein	99042-98470	573	190	100	
ORF107	ORF106	IMV membrane protein	99310-99107	204	67	100	
ORF108	ORF107	phosphorylated IMV membrane protein	99679-99392	288	95	100	
ORF109	ORF108	nonessential hydrophobic IV and IMV membrane protein	99857-99696	162	53	100	
ORF110	ORF109	late protein	100134-99847	288	95	100	
ORF111	ORF110	soluble myristylprotein	101251-100118	1134	377	100	
ORF112	ORF111	IMV membrane protein	101857-101267	591	196	100	
ORF113	ORF112	helicase	101872-103314	1443	480	100	
ORF114	ORF113	maturation protein	103519-103295	225	74	100	

ORF115	ORF114	entry-fusion complex component	103867-103520	348	115	100	
ORF116	ORF115	DNA polymerase processivity factor	103866-105158	1293	430	100	
ORF117	ORF116	Holliday junction endonuclease	105127-105633	507	168	100	
ORF118	ORF117	45kDa large subunit of intermediate transcription factor	105658-106815	1158	385	100	
ORF119	ORF118	DNA-dependent RNA polymerase subunit	106846-110316	3471	1156	100	
ORF120	ORF119	IMV surface protein	110776-110330	447	148	100	
ORF121	ORF120	entry-fusion complex component	111199-110777	423	140	100	
ORF122	ORF121	DNA-dependent RNA polymerase	112108-111200	909	302	100	
ORF123	ORF122	IMV protein	112301-112077	225	74	100	
ORF124	ORF123	hypothetical protein	112454-112329	126	41	100	
ORF125	ORF124	putative DNA packaging enzyme	113244-112480	765	254	100	
ORF126	ORF125	EEV membrane phosphoglycoprotein	113376-113966	591	196	100	
ORF127	ORF126	EEV glycoprotein	113996-114511	516	171	100	
ORF128	ORF127	MHC class II presentation inhibitor	114539-115114	576	191	100	
ORF129	ORF128	hypothetical protein	115151-116017	867	288	100	
ORF130	ORF129	hypothetical protein	116075-116620	546	181	100	
ORF131	ORF130	hypothetical protein	116631-117452	822	273	100	

ORF132	ORF131	CD47-like putative membrane protein	118358-117456	903	300	100	
ORF133	ORF132	hypothetical protein	118456-118827	372	123	100	
ORF134	ORF133	hypothetical protein	118896-119141	246	81	100	
ORF135	ORF134	inactive Cu-Zn superoxide dismutase-like protein	119197-119682	486	161	100	
ORF136	ORF135	hypothetical protein	119717-120247	531	176	100	
ORF137	ORF136	DNA ligase	120283-121956	1674	557	100	
ORF138	ORF137	poxvirus B22R superfamily protein	122,049-128,186	6138	2045	100	
ORF139	ORF138	IFN-alpha|beta-receptor-like secreted glycoprotein	128256-129338	1083	360	100	
ORF140	ORF139	Toll|IL-receptor-like protein	129386-129847	462	153	100	
ORF141	ORF140	hypothetical protein	129913-130920	1008	335	100	
ORF142	ORF141	hypothetical protein	130950-131510	561	186	100	
ORF143	ORF142	Ser|Thr kinase	131549-132466	918	305	100	
ORF144	ORF143	E3 ubiquitin-protein ligase p28-like protein	132498-133220	723	240	100	
ORF145	ORF144	hypothetical protein	133269-133946	678	225	100	
ORF146	ORF145	virokine	133948-134352	405	134	100	
ORF147	ORF146	putative tyrosine kinase	134389-135297	909	302	99.7	
ORF148	ORF147	kelch-like protein	135466-136278	813	270	100	
ORF149	ORF148	kelch-like protein	136269-137111	843	280	100	
ORF150	ORF149	hypothetical protein	137157-139061	1905	634	100	

ORF151	ORF150	phospholipase-D-like protein	139190-140431	1242	413	100	
ORF152	ORF151	hypothetical protein	140492-141988	1497	498	100	
ORF153	ORF152	hypothetical protein	142036-143379	1344	447	100	
ORF154	ORF153	serine protease inhibitor-like SPI-2	143400-144413	1014	337	100	
ORF155	ORF154	hypothetical protein	144452-144937	486	161	100	
ORF156	ORF155	kelch-like protein	144979-146631	1653	550	100	
ORF157	ORF156	hypothetical protein	146698-148167	1470	489	99.8	
ORF158	ORF157	hypothetical protein	148212-149126	915	304	25	
ORF159	ORF158	hypothetical protein	148341-149126	786	261	90.8	
ORF160	ORF159	hypothetical protein	149376-149771	396	131	100	
ORF161	ORF160	hypothetical protein	149842-150321	480	159	100	
ORF162		hypothetical protein	150,293-150,568	276	91	-	Unique

Supplementary Table 2: Open reading frames of LSDV (L3/2024)

ORF (LSDV_L3/2024)	ORF (LSDV-isolate LSDV-29)	Product	Genome coordinate_LSDV_L3/2024	Nucleotide	AA	% Identity	Note
ORF1		hypothetical protein	326-60	267	88	-	Unique
ORF2	ORF1	hypothetical protein	777-298	480	159	100	
ORF3	ORF2	hypothetical protein	1243-848	396	131	100	
ORF4	ORF3	hypothetical protein	2215-1493	723	240	100	
ORF5	ORF4	IL-10-like protein	2637-3149	513	170	99.4	
ORF6	ORF5	putative IL-1-beta-inhibitor	3855-3160	696	231	100	
ORF7	ORF6	putative IL-1 receptor antagonist	4944-3877	1068	355	100	

ORF8	ORF7	soluble interferon-gamma receptor-like protein	5855-5028	828	275	100	
ORF9	ORF8	hypothetical protein	6580-5888	693	230	100	
ORF10	ORF9	putative E3 ubiquitin ligase	7122-6634	489	162	98.2	
ORF11	ORF10	putative G-protein coupled chemokine receptor	8299-7166	1134	377	99	
ORF12	ORF11	ankyrin repeat protein	9040-8405	636	211	99.1	
ORF13	ORF12	IL-1 receptor-like protein	10104-9079	1026	341	100	
ORF14	ORF13	eIF2alpha-like PKR inhibitor	10433-10164	270	89	100	
ORF15	ORF14	putative IL-18 binding protein	10905-10420	486	161	100	
ORF16	ORF15	EGF-like growth factor	11213-10944	270	89	100	
ORF17	ORF16	antiapoptotic protein	11734-11204	531	176	100	
ORF18	ORF17	dUTPase	12213-11773	441	146	100	
ORF19	ORF18	kelch-like protein	13133-12261	873	290	100	
ORF20	ORF19	kelch-like protein	13971-13276	696	231	85.1	
ORF21	ORF20	ribonucleotide reductase small subunit	15001-14036	966	321	100	
ORF22	ORF21	hypothetical protein	15302-15042	261	86	100	
ORF23	ORF22	hypothetical protein	15687-15343	345	114	98.2	
ORF24	ORF23	hypothetical protein	16119-15901	219	72	100	
ORF25	ORF24	S-S bond formation pathway protein	16847-16197	651	216	100	

ORF26	ORF25	putative Ser|Thr protein kinase	18168-16825	1344	447	100	
ORF27	ORF26	Pox F11 superfamily protein	18666-18328	339	112	71.2	
ORF28	ORF27	Pox F11 superfamily protein	19113-18790	324	107	100	
ORF29	ORF28	hypothetical protein	21038-19122	1917	638	100	
ORF30	ORF29	palmitylated EEV membrane protein	22157-21045	1113	370	100	
ORF31	ORF30	hypothetical protein	22330-22184	147	48	100	
ORF32	ORF31	Pox F15 superfamily protein	22796-22359	438	145	100	
ORF33	ORF32	Pox F16 superfamily protein	23532-22873	660	219	100	
ORF34	ORF33	putative DNA-binding phosphoprotein	23606-23920	315	104	100	
ORF35	ORF34	poly-A polymerase catalytic subunit	25348-23924	1425	474	100	
ORF36	ORF35	hypothetical protein	27552-25345	2208	735	99.9	
ORF37	ORF36	double-stranded RNA binding protein	28097-27564	534	177	100	
ORF38	ORF37	DNA-dependent RNA polymerase subunit	28764-28159	606	201	99	
ORF39	ORF38	abundant component of virosome	28847-29971	1125	374	99.5	
ORF40	ORF39	hypothetical protein	29980-31680	1701	566	100	
ORF41	ORF40	membrane protein	31687-32487	801	266	100	
ORF42	ORF41	DNA polymerase	35516-32484	3033	1010	100	
ORF43	ORF42	sulfhydryl oxidase	35550-35837	288	95	100	

ORF44	ORF43	virion core protein	36226-35834	393	130	100	
ORF45	ORF44	hypothetical protein	38267-36213	2055	684	99.9	
ORF46	ORF45	DNA-binding core protein	39317-38373	945	314	100	
ORF47	ORF46	viral morphogenesis protein	39542-39324	219	72	100	
ORF48	ORF47	ssDNA-binding phosphoprotein	40373-39543	831	276	100	
ORF49	ORF48	IMV protein	40654-40418	237	78	100	
ORF50	ORF49	telomere mismatch binding protein	41856-40672	1185	394	100	
ORF51	ORF50	viral core cysteine proteinase	43150-41849	1302	433	100	
ORF52	ORF51	RNA-helicase	43156-45186	2031	676	100	
ORF53	ORF52	putative metalloproteinase	46973-45183	1791	596	100	
ORF54	ORF53	entry-fusion complex component	47302-46970	333	110	100	
ORF55	ORF54	late transcription elongation factor	47296-47964	669	222	100	
ORF56	ORF55	thioredoxin-like protein	48308-47928	381	126	100	
ORF57	ORF56	Fen1-like nuclease	48311-49624	1314	437	100	
ORF58	ORF57	DNA-dependent RNA polymerase subunit	49625-49816	192	63	100	
ORF59	ORF58	hypothetical protein	49816-50340	525	174	100	
ORF60	ORF59	virion structural protein	51475-50354	1122	373	100	
ORF61	ORF60	late transcription factor	51505-52287	783	260	100	

ORF62	ORF61	myristylprotein	52314-53324	1011	336	100	
ORF63	ORF62	IMV membrane protein	53325-54062	738	245	100	
ORF64	ORF63	crescent formation protein	54100-54378	279	92	98.9	
ORF65	ORF64	early transcription protein	55344-54388	957	318	100	
ORF66	ORF65	core protein	55369-56130	762	253	100	
ORF67	ORF66	putative membrane protein	56146-56541	396	131	100	
ORF68	ORF67	virion protein	56498-56941	444	147	100	
ORF69	ORF68	thymidine kinase	56969-57502	534	177	100	
ORF70	ORF69	host-range protein	57574-58167	594	197	99.5	
ORF71	ORF70	multifunctional poly-A polymerase regulatory subunit	58227-59228	1002	333	100	
ORF72	ORF71	DNA-dependent RNA polymerase subunit	59143-59700	558	185	100	
ORF73	ORF72	late 16kDa putative membrane protein	60107-59706	402	133	100	
ORF74	ORF73	DNA-dependent RNA polymerase subunit	60175-64032	3858	1285	100	
ORF75	ORF74	Tyr|Ser protein phosphatase	64552-64037	516	171	100	
ORF76	ORF75	entry-fusion complex component	64568-65140	573	190	100	
ORF77	ORF76	IMV heparin binding surface protein	66105-65137	969	322	100	
ORF78	ORF77	hypothetical protein	68531-66135	2397	798	99.9	
ORF79	ORF78	late transcription factor	68676-69347	672	223	100	

ORF80	ORF79	topoisomerase type IB	69389-70342	954	317	100	
ORF81	ORF80	virion morphogenesis protein	70363-70806	444	147	100	
ORF82	ORF81	large subunit of mRNA capping enzyme	70837-73365	2529	842	100	
ORF83	ORF82	virion core protein	73794-73327	468	155	100	
ORF84	ORF83	virion core protein	73796-74533	738	245	100	
ORF85	ORF84	uracil-DNA glycosylase	74530-75186	657	218	100	
ORF86	ORF85	NTPase	75229-77589	2361	786	100	
ORF87	ORF86	70kDa small subunit of early transcription factor	77586-79493	1908	635	100	
ORF88	ORF87	DNA-dependent RNA polymerase subunit	79518-80009	492	163	100	
ORF89	ORF88	mRNA decapping enzyme	80050-80691	642	213	100	
ORF90	ORF89	mRNA decapping enzyme	80,691-81,023	333	110	100	
ORF91	ORF89	mRNA decapping enzyme	81187-81453	267	88	34.8	
ORF92	ORF90	ATPase nucleoside triphosphate phosphohydrolase-I	83366-81459	1908	635	100	
ORF93	ORF91	small subunit of mRNA capping enzyme	84256-83393	864	287	99.7	
ORF94	ORF92	rifampicin target	85946-84297	1650	549	100	

ORF95	ORF93	late transcription factor	86425-85973	453	150	99.3	
ORF96	ORF94	putative late transcription factor	87153-86455	699	232	100	
ORF97	ORF95	S-S bond formation pathway protein	87377-87150	228	75	100	
ORF98	ORF96	hypothetical protein	89371-87386	1986	661	99.7	
ORF99	ORF97	putative virion core protein	89981-89496	486	161	100	
ORF100	ORF98	DNA-dependent RNA polymerase subunit	90022-90531	510	169	99.4	
ORF101	ORF99	virion core protein	91655-90528	1128	375	100	
ORF102	ORF100	82kDa large subunit of early transcription factor	93820-91676	2145	714	100	
ORF103	ORF101	32kDa small subunit of transcription factor	93877-94749	873	290	100	
ORF104	ORF102	IMV membrane protein	95009-94773	237	78	100	
ORF105	ORF103	hypothetical protein	97724-95010	2715	904	100	
ORF106	ORF104	viral membrane formation protein	97739-98692	954	317	100	
ORF107	ORF105	core protein	99261-98689	573	190	98.9	
ORF108	ORF106	IMV membrane protein	99529-99326	204	67	100	
ORF109	ORF107	phosphorylated IMV membrane protein	99898-99611	288	95	100	
ORF110	ORF108	nonessential hydrophobic IV and IMV membrane protein	100076-99915	162	53	100	

ORF111	ORF109	late protein	100353-100066	288	95	100	
ORF112	ORF110	soluble myristylprotein	101470-100337	1134	377	99.7	
ORF113	ORF111	IMV membrane protein	102076-101486	591	196	100	
ORF114	ORF112	helicase	102091-103533	1443	480	99.6	
ORF115	ORF113	maturation protein	103738-103514	225	74	100	
ORF116	ORF114	entry-fusion complex component	104086-103739	348	115	100	
ORF117	ORF115	DNA polymerase processivity factor	104085-105377	1293	430	100	
ORF118	ORF116	Holliday junction endonuclease	105346-105852	507	168	100	
ORF119	ORF117	45kDa large subunit of intermediate transcription factor	105877-107034	1158	385	100	
ORF120	ORF118	DNA-dependent RNA polymerase subunit	107065-110535	3471	1156	100	
ORF121	ORF119	IMV surface protein	110995-110549	447	148	100	
ORF122	ORF120	entry-fusion complex component	111418-110996	423	140	100	
ORF123	ORF121	DNA-dependent RNA polymerase	112330-111419	912	303	99.7	
ORF124	ORF122	IMV protein	112523-112299	225	74	100	
ORF125	ORF123	hypothetical protein	112676-112551	126	41	100	
ORF126	ORF124	putative DNA packaging enzyme	113466-112702	765	254	100	
ORF127	ORF125	EEV membrane phosphoglycoprotein	113598-114188	591	196	100	
ORF128	ORF126	EEV glycoprotein	114221-114736	516	171	100	

ORF129	ORF127	MHC class II presentation inhibitor	114764-115339	576	191	100	
ORF130	ORF128	hypothetical protein	115376-116242	867	288	100	
ORF131	ORF129	hypothetical protein	116301-116846	546	181	100	
ORF132	ORF130	hypothetical protein	116857-117678	822	273	99.6	
ORF133	ORF131	CD47-like putative membrane protein	118587-117682	906	301	99.3	
ORF134	ORF132	hypothetical protein	118685-119056	372	123	99.2	
ORF135	ORF133	hypothetical protein	119125-119370	246	81	100	
ORF136	ORF134	inactive Cu-Zn superoxide dismutase-like protein	119426-119911	486	161	100	
ORF137	ORF135	hypothetical protein	119946-120476	531	176	100	
ORF138	ORF136	DNA ligase	120512-122185	1674	557	99.8	
ORF139	ORF137	poxvirus B22R superfamily protein	122340-128417	6078	2025	100	
ORF140	ORF138	IFN-alpha|beta-receptor-like secreted glycoprotein	128487-129569	1083	360	100	
ORF141	ORF139	Toll|IL-receptor-like protein	129617-130078	462	153	100	
ORF142	ORF140	hypothetical protein	130144-131151	1008	335	100	
ORF143	ORF141	hypothetical protein	131181-131741	561	186	100	
ORF144	ORF142	Ser|Thr kinase	131780-132697	918	305	100	
ORF145	ORF143	E3 ubiquitin-protein ligase p28-like protein	132729-133451	723	240	98.8	
ORF146	ORF144	hypothetical protein	133500-134174	675	224	99.6	
ORF147	ORF145	virokine	134176-134580	405	134	100	

ORF148	ORF146	putative tyrosine kinase	134617-135525	909	302	99.7	
ORF149	ORF147	kelch-like protein	135694-137346	1653	550	47.4	
ORF150	ORF148	kelch-like protein	136495-137346	852	283	98.6	
ORF151	ORF149	hypothetical protein	137384-139288	1905	634	99.8	
ORF152	ORF150	phospholipase-D-like protein	139417-140658	1242	413	100	
ORF153	ORF151	hypothetical protein	140,673-142,217	1545	514	100	
ORF154	ORF152	hypothetical protein	142265-143608	1344	447	100	
ORF155	ORF153	serine protease inhibitor-like SPI-2	143629-144642	1014	337	99.7	
ORF156	ORF154	hypothetical protein	144681-145166	486	161	100	
ORF157	ORF155	kelch-like protein	145208-146863	1656	551	99.6	
ORF158	ORF156	hypothetical protein	146930-148399	1470	489	99.8	
ORF159	ORF157	hypothetical protein	148444-148698	255	84	72.7	
ORF160	ORF158	hypothetical protein	148916-149638	723	240	100	
ORF161	ORF159	hypothetical protein	149888-150283	396	131	100	
ORF162	ORF160	hypothetical protein	150354-150833	480	159	100	
ORF163		hypothetical protein	150,805-151,071	267	88	-	Unique
